# Development of a Property-Guided HS-SPME-GC-MS Method for Broad-Range Qualitative Screening of Potentially Hazardous VOCs in Recycled Polypropylene

**DOI:** 10.1021/acspolymersau.6c00124

**Published:** 2026-08-24

**Authors:** Wei Jiang, Huijun Yi, Caleb Chatfield, Chloe Higdon, Nathan Mancos, Shaoyang Liu

**Affiliations:** † Center for Materials and Manufacturing Sciences, Departments of Chemistry and Physics, 8025 Troy University, Troy, Alabama 36082, United States; ‡ Department of Mathematics and Statistics, Troy University, Troy, Alabama 36082, United States

**Keywords:** recycled plastic, volatile organic compound, headspace solid-phase microextraction, GC-MS, response surface methodology

## Abstract

The increasing use of recycled plastics has raised concerns regarding the potential emission of hazardous volatile organic compounds (VOCs) during recycling and in subsequent applications. However, systematic analytical methods for VOC analysis in real recycled plastic materials remain limited due to the complex and diverse composition of emitted VOCs. In this study, a qualitative analytical method based on headspace solid-phase microextraction coupled with gas chromatography–mass spectrometry (HS-SPME-GC-MS) was developed for characterizing potentially hazardous VOCs in recycled polypropylene (rPP). A total of 622 candidate contaminants associated with recycled plastics were compiled. After screening by NFPA health hazard rating and volatility, 274 potentially hazardous VOCs were retained, and 27 representative compounds were selected to cover broad vapor-pressure and polarity ranges. Five SPME fiber coatings were evaluated, among which DVB/C-WR/PDMS provided the broadest detection coverage, detecting 24 of the 27 representative compounds. Response surface methodology based on rotatable central composite designs was then used to optimize SPME temperature, incubation time, and extraction time. SPME temperature was the dominant factor affecting extraction efficiency, and that vapor pressure was the primary physicochemical determinant of extraction behavior. High- and medium-vapor-pressure VOCs were most efficiently extracted under relatively moderate-temperature conditions, with an optimized SPME temperature of 68.7 °C, incubation time of 5.5 min, and extraction time of 10.4 min. In contrast, low-vapor-pressure VOCs required higher temperatures to promote transfer from the polymer matrix to the headspace, and 110 °C was identified as a practical compromise. Using the optimized method, aliphatic hydrocarbons, aromatic hydrocarbons, oxygenated compounds, and lindane were detected in industrial rPP samples, indicating multiple potential contamination sources in recycled plastic streams. This study provides a systematic HS-SPME-GC-MS strategy for broad qualitative screening of potentially hazardous VOCs in rPP and may support quality control, emission assessment, and process optimization in plastic recycling.

## Graphical abstract

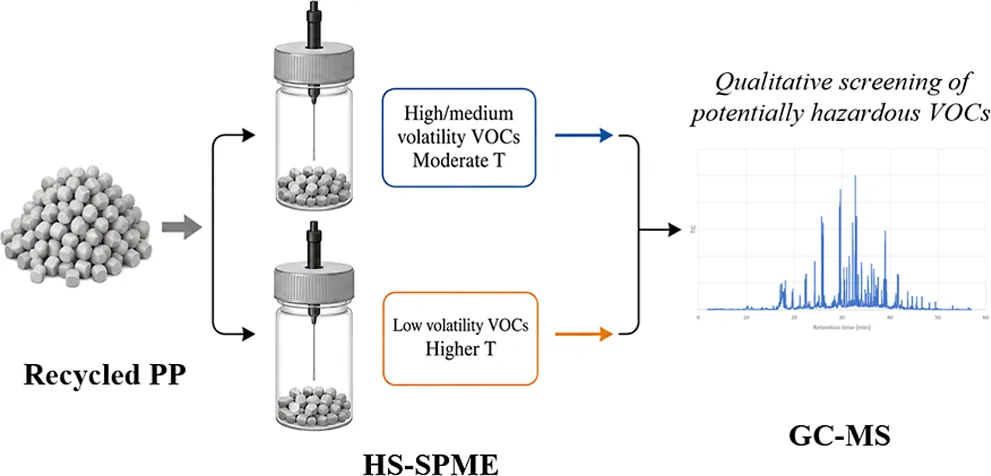

## 1 Introduction

Plastic waste has become a major source of environmental pollution, posing persistent threats to both terrestrial and aquatic ecosystems worldwide.^1,2^ Since the mid-20th century, the rapid growth in plastic production and consumption has resulted in a substantial accumulation of plastic waste. Global plastic production reached approximately 8300 million metric tons (Mt) in 2017, while plastic waste generation exceeded 6300 Mt.^3^ Recycling is widely recognized as an effective strategy to reduce plastic pollution and conserve natural resources.^4,5^ However, despite increasing awareness and policy efforts, the global plastic recycling rate remains low. After accounting for processing losses, only approximately 9% of plastic waste is ultimately recycled, whereas the majority is landfilled, incinerated, or mismanaged, leading to long-term environmental contamination.^6^

Improving recycling rates requires not only the expansion of collection and processing capacity but also enhancements in the quality and safety of recycled plastic products. In recent years, the performance of recycled plastics has become a critical factor influencing their market acceptance and broader application.^7^ Mechanical recycling, which dominates current recycling systems, often leads to materials with degraded properties and undesirable sensory characteristics, limiting their use in high-value products.^8^ Among these quality-related challenges, the presence and release of volatile organic compounds (VOCs) have attracted growing attention. Recycled plastics can emit complex mixtures of VOCs originating from polymer thermo-oxidative degradation during service life and reprocessing, as well as residual additives, absorbed contaminants, and substances introduced during previous use cycles and recycling processes.^9^ These VOCs can be released during recycling operations such as washing, drying, melting, and extrusion, as well as during subsequent product use, leading to unpleasant odors and raising concerns regarding potential health risks. Recently, some VOCs detected in recycled plastics, including aromatic hydrocarbons and low-molecular-weight oxygenated compounds, are classified as hazardous substances by international authorities.^10,11^ These concerns highlight the importance of reliable VOC detection for improving the quality and safety of recycled plastics and strengthening public confidence in recycled materials. Consequently, the development of systematic and sensitive analytical methods for characterizing VOCs in recycled plastics has become a pressing need to support sustainable recycling practices and circular economy goals.

Over the past decade, substantial progress has been made in characterizing VOCs emitted from recycled plastics. Gas chromatography–mass spectrometry (GC–MS) remains the principal analytical technique because of its high separation efficiency and capacity for structural identification.^9^ Among GC-MS-based approaches, headspace solid-phase microextraction coupled with GC–MS (HS-SPME-GC-MS) has emerged as a powerful and versatile method for this application. HS-SPME is a solvent-free sample preparation method that integrates sampling, enrichment, and matrix isolation in a single step. By extracting analytes directly from the headspace, HS-SPME minimizes interference from nonvolatile matrix components and is particularly well-suited for solid polymer materials.^12,13^ The extraction efficiency of HS-SPME can be tuned by selecting appropriate fiber coatings and optimizing experimental parameters such as SPME temperature, incubation time, and extraction time, allowing coverage of VOCs with diverse physicochemical properties.^13,14^ Several studies have demonstrated the applicability of HS-SPME-GC-MS for investigating VOC emissions from recycled plastics, particularly in relation to unpleasant odors that limit material acceptance. HS-SPME-GC-MS was applied to identify odor-causing compounds in recycled polyolefin resins, while subsequent studies used similar approaches to track changes in VOC profiles during mechanical recycling and to evaluate mitigation strategies.^15–17^ However, many existing studies have primarily focused on VOCs that could cause undesirable odors or were present in high concentrations but overlooked trace-level hazardous contaminants, which could pose safety concerns for recycled plastic products. Systematic method-development approaches targeting potentially hazardous VOCs across a broad range of physicochemical properties remain scarce.

In this study, we developed and optimized practical HS-SPME-GC-MS methods for the detection of a broad range of potentially hazardous VOCs in recycled polypropylene (rPP). Relevant physicochemical property ranges were first established through an extensive survey of potentially hazardous VOCs in recycled plastics. Representative target compounds were then systematically selected to cover the broad ranges of volatility and polarity. Different SPME fiber coatings were evaluated, and key HS-SPME parameters were optimized using response surface methodology. The applicability of the optimized method was further demonstrated using industrial rPP samples. Overall, this work provides a robust analytical framework for VOC screening that can support quality assurance, emission assessment, and process optimization in plastic recycling systems.

## 2 Materials and Methods

### 2.1 Chemicals and Recycled Plastics

Virgin PP pellets (molecular weight: ∼ 250 K, diameter: ∼ 4 mm) and 27 VOCs with diverse physicochemical properties, including myristic acid, ethylene brassylate, *p*-cymene, n-pentadecane, 1-decanol, butylated hydroxytoluene (BHT), 2,4-dimethyl-1-heptene, 1-butanol, 1,3-diethylbenzene, lindane, m-xylene, toluene, 2,4-dichlorophenoxyacetic acid (2,4-D), MCPA, 4-hydroxy-3-*tert*-butylanisole (BHA), 4-(4-hydroxyphenyl)-2-butanone, tetrahydrolinalool, γ-decalactone, ethyl salicylate, 2-ethylhexanal, dimethoate, ethylene glycol, pentanamide, ε-caprolactam, ethyl acetate, 5-methylfurfural, and ethyl butanoate, were used for analytical method development and optimization. All of the above chemicals, purchased from Fisher Scientific, were of analytical grade and were used as received. Postconsumer recycled PP samples were obtained from industrial plastic recyclers. Prior to analysis, the recycled PP samples were stored in sealed containers at room temperature to minimize contamination and volatilization losses.

### 2.2 Data Collection for the Survey of Potentially Hazardous VOCs in Recycled Plastics

A comprehensive inventory of potentially hazardous VOCs associated with recycled plastics was established through a literature review and data compilation. The target compounds were compiled from three major categories: (1) VOCs previously reported in recycled plastics,^9^ (2) commonly used household and commercial pesticides and herbicides, and (3) widely used plastic additives. In total, 622 candidate compounds were included for further evaluation.

NFPA hazard ratings for these chemicals were first collected from authoritative governmental or government-supported databases, including the Occupational Safety and Health Administration (OSHA), CAMEO Chemicals, and PubChem. When ratings were not available from these databases, Safety Data Sheets (SDSs) from major chemical manufacturers (e.g., Fisher Scientific and Sigma-Aldrich) were consulted. If NFPA ratings were not explicitly provided in the SDSs, Gemini was used to predict the ratings based on hazard information reported in the SDSs. When the required hazard information remained unavailable, additional credible chemical information sources (e.g., ChemicalBook and Chemical Safety databases) were consulted, and Gemini was again used to estimate the ratings when necessary.

Among the compiled substances, 367 chemicals with NFPA health hazard ratings of 2 or more were identified as potentially hazardous contaminants. For these compounds, room-temperature vapor pressures and octanol–water partition coefficients (logP) were collected to characterize their volatility and polarity, respectively. Vapor pressure data were obtained using the same tiered data-collection procedure described above. LogP values were obtained from PubChem and calculated using the XLogP3 (version 3.0) method. Finally, 27 representative VOCs were systematically selected to provide broad coverage of volatility and polarity ranges for analytical method development.

### 2.3 Response Surface Methodology and Experimental Design

Response surface methodology (RSM) based on a three-factor rotatable central composite design (CCD) was employed to evaluate the effects of SPME temperature, incubation time, and extraction time on the SPME extraction efficiency. The SPME temperature refers to the vial temperature maintained during both the pre-extraction incubation period and the subsequent fiber exposure/extraction period. The CCD consisted of 20 experimental runs, including 8 factorial points (2^3^), 6 axial (star) points (2k), and 6 center points for the estimation of pure error and assessment of model adequacy. Rotatability was ensured by setting the axial distance to α = 1.682 (k = 3).

To keep the experimental domain within feasible ranges, the CCDs were coded so that the axial points (±α) corresponded to the practical limits of each factor: 60–120 °C and 3–11 min for the first CCD, and 30–90 °C and 3–11 min for the second CCD. The experimental factors were transformed into coded variables (*x*_*i*_) using

$$x_{i}=\frac{X_{i}-X_{0}}{\Delta X}$$

where *X*_*i*_ is the actual value of the factor, *X*_0_ is the center point, and Δ*X* is the step change corresponding to one coded unit.

The experimental data were fitted to a second-order polynomial model

$$Y=\beta_{0}+\sum_{i=1}^{3}\beta_{i}x_{i}+\sum_{i=1}^{3}\beta_{ii}x_{i}^{2}+\sum_{i<j}^{3}\beta_{ij}x_{i}x_{j}$$

For each CCD experimental run, HS-SPME-GC-MS analysis was performed using virgin PP pellets spiked with a mixture of the 27 representative VOCs. The chromatographic peak areas of the target compounds, determined using selected quantifier ions, were used as dependent variables for model fitting. RSM was applied to evaluate the individual and interactive effects of the three HS-SPME parameters and to identify the optimal extraction conditions.

### 2.4 HS-SPME-GC-MS Analysis

HS-SPME-GC-MS analyses were performed using an Agilent 8890 gas chromatograph coupled with an Agilent 7250 GC/Q-TOF mass spectrometer (Agilent Technologies, USA). VOCs were separated on a low-polarity capillary column suitable for volatile and semivolatile organic compounds (DB-5 ms, 30 m × 0.25 mm × 0.25 μm).

A VOC-spiked PP model sample was prepared to simulate contamination in recycled plastics. Briefly, a mixture of the 27 representative VOCs was prepared by mixing 50 mg of each solid analyte or 50 μL of each liquid analyte into a 20 mL glass vial. The mixture was homogenized by stirring and heated at 70 °C for 3 min to ensure complete dissolution and mixing. For the first CCD experiments, 200 g of virgin PP pellets were weighed in a 1 L glass bottle, and 10 μL of the VOC mixture was introduced. The VOC spike was deposited onto a piece of Kimwipe to avoid direct contact with the PP pellets. Then, the glass bottle was sealed, manually mixed 2–3 times per day, and stored under room temperature for 1 week prior to analysis to ensure uniform distribution of the VOCs on the PP pellets. For the second CCD experiments, the mass of the PP pellets was increased to 500 g and the volume of the VOC mixture was reduced to 2.0 μL to prepare a PP model sample with less contaminations.

For HS-SPME extraction, 1.0 g aliquots of the contaminated PP pellets were transferred into 20 mL headspace vials and sealed with PTFE/silicone septa. After incubation under specified conditions, the SPME fiber was exposed to the vial headspace for VOC extraction and subsequently thermally desorbed in the GC inlet. In this study, five SPME fiber coatings, including DVB/C-WR/PDMS, C-WR/PDMS, PA, PDMS, and PEG were investigated. For the recycled PP samples, each sample was homogenized before subsampling, and a single 1.0 g aliquot was analyzed under each of the two optimized HS-SPME conditions.

Gas chromatographic conditions were as follows: inlet temperature, 250 °C; injection mode, splitless; carrier gas, helium at a constant flow rate of 1.0 mL/min. The oven temperature program was set to 40 °C (held for 3 min), ramped to 250 °C at 4 °C/min, and held for 1.5 min.

Mass spectrometric detection was conducted using electron ionization (EI) at 70 eV. The ion source temperature was set to 230 °C, and the quadrupole temperature was 150 °C. Data were acquired in full-scan mode over an *m*/*z* range of 35–500, with targeted extraction used for improved identification and data processing of specific VOCs.

### 2.5 Data Analysis Methods

GC–MS data were processed using Agilent MassHunter software. VOCs were identified based on retention times, characteristic mass spectral fragments, and comparisons with reference standards and mass spectral libraries. For each target VOC, one quantifier ion and two qualifier ions were selected to ensure reliable identification.

For response surface analysis, peak areas of the 27 representative VOCs were integrated using the selected quantifier ions and used as response variables. Statistical modeling, analysis of variance (ANOVA), and response surface visualization were performed using dedicated RSM software (Design-Expert version 25, Stat-Ease, USA). Model fitting was evaluated based on the coefficient of determination (R^2^), adjusted R^2^, predicted R^2^, and the F value of the model parameters. The optimal HS-SPME conditions were determined by maximizing the responses.

Correlation analysis was conducted using R software (version 4.5.3).

## 3 Results and Discussion

### 3.1 Selection of Representative Hazardous VOCs

An extensive survey was conducted to identify potentially hazardous VOCs associated with recycled plastics. A total of 622 potential contaminants were compiled from three categories: VOCs previously reported in recycled plastics (n = 435), commonly used household and commercial pesticides and herbicides (n = 114), and widely used plastic additives (n = 73). NFPA health hazard ratings for all candidates were obtained from government agencies, major chemical manufacturers, and other credible sources (Table S1). Among the 622 evaluated chemicals, 14 received a hazard rating of 4, 96 a rating of 3, 257 a rating of 2, 149 a rating of 1, and 106 a rating of 0. Chemicals with NFPA health hazard ratings ≥2 (n = 367) were classified as potentially hazardous contaminants.

Vapor pressure at room temperature and logP values were subsequently compiled for these 367 chemicals as indicators of volatility and polarity, respectively. Sixty-one chemicals were excluded due to the absence of reliable data. An additional 32 chemicals were excluded because their vapor pressures were below 1 × 10^–7^ mmHg, indicating negligible volatility and limited suitability for extraction by HS-SPME. The remaining 274 chemicals were therefore considered as potentially hazardous VOCs in recycled plastics for this study (Table S2).

These compounds were categorized based on volatility and polarity, which are the two key parameters influencing SPME extraction efficiency, to ensure systematic coverage of their diverse physicochemical properties. The one-third and two-third quantiles of each parameter were used to divide the data set into three levels (high, medium, and low), resulting in a 3 × 3 classification matrix comprising nine physicochemical groups (Figure 1). Three representative VOCs were randomly selected from each group, yielding 27 compounds in total, for optimization of the HS-SPME-GC-MS analytical method (Table S3).

**Figure 1:**
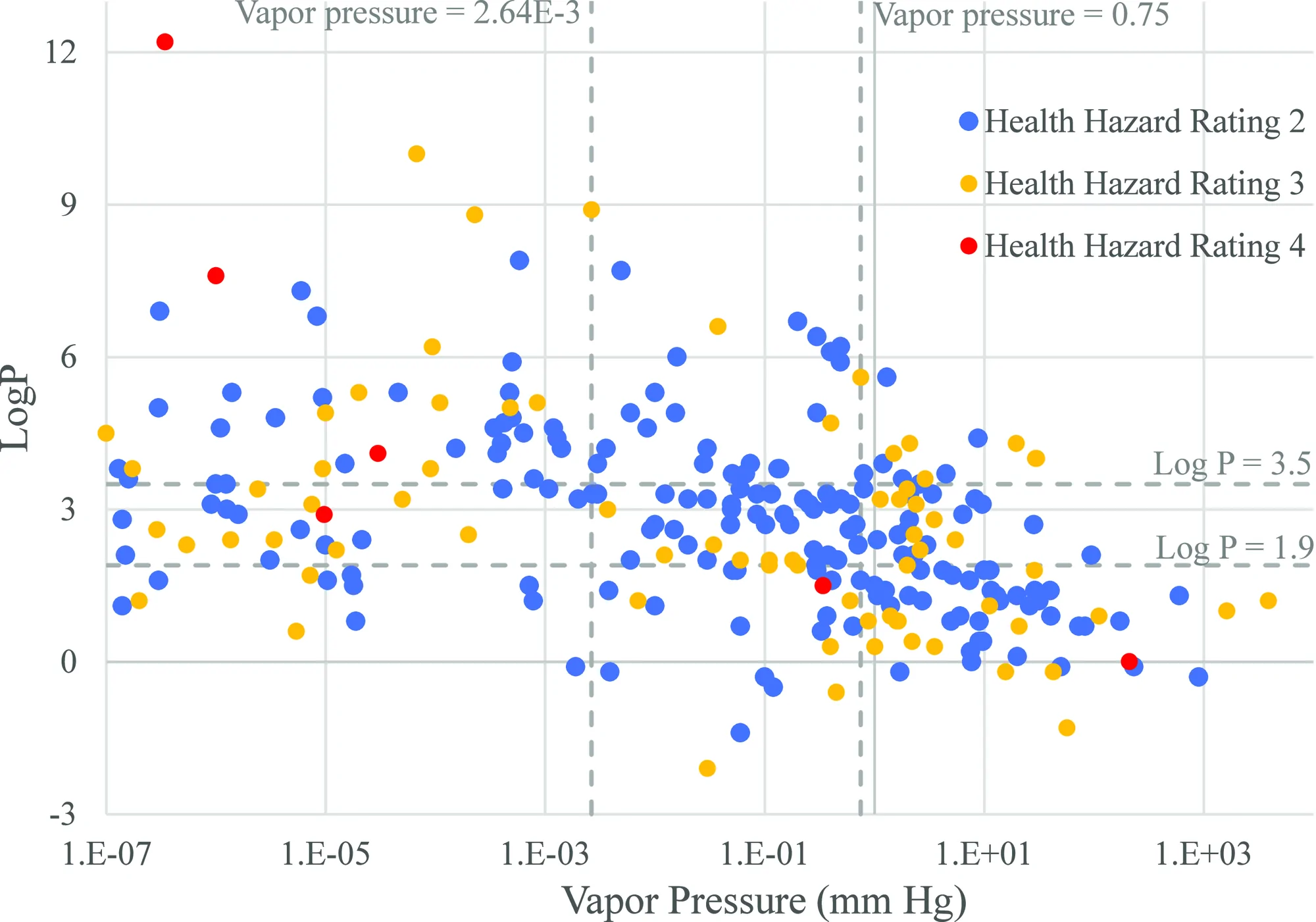
Classification of candidate VOCs based on vapor pressure and polarity.

The NFPA health hazard rating was used in this study as a practical screening criterion to prioritize compounds for analytical method development rather than as a definitive assessment of exposure or risk. Actual emission and exposure potential in recycled-plastic systems may also depend on analyte concentration, volatility, retention within the polymer matrix, processing and end-use conditions, and exposure duration. Consequently, compounds with NFPA health hazard ratings ≥2 may have limited emission potential if they are weakly volatile or strongly retained within the polymer, whereas some lower-rated VOCs may still be relevant when present at high concentrations or readily emitted.

### 3.2 Effects of SPME Fiber Coating Type on Extraction Efficiency

Pure standards of the 27 selected representative VOCs were analyzed individually using the HS-SPME-GC-MS method to determine their retention times (RTs) and to select the corresponding mass spectral ions, including the quantifier ion (#1) and qualifier ions (#2 and #3). The results are summarized in Table 1, together with the room-temperature vapor pressure and logP values.

**Table 1:** Retention Times, Physicochemical Properties, and Selected Mass Spectral Ions of Representative VOCs

						MS (*m*/*z*)		
Group	Chemical name	CAS #	Log*P*	VP (mmHg)^a^	RT (min)	1	2	3
1	2,4-dimethyl-1-heptene	19549–87–2	4.4	8.80 × 10^0^	11.84	43	70	55
	1,3-diethylbenzene	141–93–5	3.9	1.20 × 10^0^	18.86	105	134	91
	*p*-cymene	99–87–6	4.1	1.46 × 10^0^	19.41	119	134	91
2	toluene	108–88–3	2.7	2.10 × 10^1^	9.12	91	92	65
	*m*-xylene	108–38–3	3.2	8.29 × 10^0^	13.03	91	106	77
	2-ethylhexanal	123–05–7	2.6	2.00 × 10^0^	16.48	57	72	41
3	ethyl acetate	141–78–6	0.7	9.32 × 10^1^	5.36	43	61	70
	1-butanol	71–36–3	0.9	7.00 × 10^0^	6.08	56	31	41
	ethyl butanoate	105–54–4	1.3	1.55 × 10^1^	10.33	71	43	88
4	1-undecene	821–95–4	6.2	4.93 × 10^–1^	22.6	55	69	43
	1-decanol	112–30–1	4.6	8.51 × 10^–3^	28.7	55	43	69
	butylated hydroxytoluene (BHT)	128–37–0	5.3	1.00 × 10^–2^	37.06	205	220	57
5	tetrahydrolinalool	78–69–3	3.3	1.15 × 10^–1^	22.25	73	69	55
	ethyl salicylate	118–61–6	3	5.40 × 10^–2^	28.84	120	92	166
	γ-decalactone	706–14–9	2.7	5.12 × 10^–3^	35.47	85	55	99
6	ethylene glycol	107–21–1	–1.4	6.00 × 10^–2^	7.8	43	42	61
	5-methylfurfural	620–02–0	0.7	6.40 × 10^–1^	16.86	110	109	53
	pentanamide	626–97–1	0.7	6.00 × 10^–2^	20.3	59	44	72
7	myristic acid	544–63–8	5.3	1.40 × 10^–6^	43.86	129	185	228
	lindane	58–89–9	3.7	4.20 × 10^–5^	44.28	219	181	109
	ethylene brassylate	105–95–3	4.2	1.54 × 10^–4^	50.83	55	41	98
8	4-hydroxy-3-*tert*-butylanisole (BHA)	121–00–6	3.2	2.48 × 10^–3^	35.99	165	137	180
	MCPA	94–74–6	2.8	1.46 × 10^–6^	39.32	141	200	77
	2,4-dichlorophenoxyacetic acid (2,4-D)	94–75–7	2.8	1.05 × 10^–7^	43.76	162	164	175
9	ε-caprolactam	105–60–2	–0.1	1.90 × 10^–3^	27.95	55	85	113
	4-(4-hydroxyphenyl)-2-butanone	5471–51–2	1.5	7.16 × 10^–4^	37.99	107	164	94
	Dimethoate	60–51–5	0.7	1.88 × 10^–5^	43.32	87	93	125

a Note: VP denotes vapor pressure at 25 °C.

The performance of different SPME fiber coatings was evaluated under the following conditions: SPME temperature of 90 °C, incubation time of 7 min, and extraction time of 7 min. The target VOCs were classified into three categories according to their chromatographic peak areas. VOCs with peak areas larger than 1 × 10^6^ were considered readily detectable, those with areas between 1 × 10^4^ and 1 × 10^6^ were classified as moderately detectable, and VOCs showing no obvious peaks (area <1 × 10^4^) were considered nondetectable. As summarized in Table 2 and S4, most VOCs with high or medium vapor pressures (Groups 1–6) were readily detectable, whereas the detection of low-vapor-pressure VOCs (Groups 7–9) was much more challenging, indicating that VOC volatility was a critical factor affecting SPME extraction efficiency under the tested conditions. Among the tested fibers, the DVB/C-WR/PDMS fiber exhibited the best overall performance, with the highest number of detectable VOCs (24 compounds) and the greatest number of readily detectable VOCs (19 compounds). Therefore, the DVB/C-WR/PDMS fiber was selected for subsequent experiments, as it provided the most balanced and comprehensive detection performance across VOCs with varying volatility and polarity.

**Table 2:** Performance of Different SPME Fibers

Fiber coating type	DVB/C-WR/PDMS	C-WR/PDMS	PA	PDMS	PEG
No. of readily detectable VOCs	19	18	17	18	18
No. of moderately detectable VOCs	5	5	7	6	5
No. of VOCs detected in total	24	23	24	24	23

Among the 27 VOCs evaluated, three compounds, i.e., ethylene glycol, MCPA, and 2,4-D, were not detected using any of the tested SPME fibers. The nondetection of MCPA and 2,4-D is primarily attributable to their very low volatility, as indicated by their extremely low vapor pressures: 1.46 × 10^–6^ mmHg for MCPA and 1.05 × 10^–7^ mmHg for 2,4-D. These low vapor pressures substantially limit their partitioning from the PP matrix into the headspace, thereby reducing their availability for extraction by the SPME fiber. In contrast, the nondetection of ethylene glycol is likely associated with its very high polarity and hydrophilicity, as reflected by its negative logP value (−1.4), the only negative logP among the 27 selected VOCs. This property may reduce its affinity for the tested fiber coatings and reduce extraction efficiency under the applied HS-SPME conditions. Overall, these results indicate that the proposed HS-SPME-GC-MS method may have limitations for analytes with extremely low volatility or very high polarity/hydrophilicity.

### 3.3 Optimization of the HS-SPME-GC-MS Method

Preliminary experiments were first conducted using PP samples spiked with the 27 representative VOCs to estimate suitable SPME parameter ranges. Based on these results, the first CCD was designed with an SPME temperature range of 60–120 °C and incubation and extraction times of 3–11 min. These experiments indicated that increasing the incubation or extraction time toward 11 min did not consistently improve the responses of many representative VOCs and, in some cases, resulted in decreased responses. Therefore, the range of 3–11 min was selected to identify practical conditions for rapid qualitative screening rather than to ensure extraction equilibrium. The actual and coded experimental parameters are listed in Table 3. A total of 20 response surface experiments were performed using the spiked PP samples. A representative total ion chromatogram (TIC) obtained at the center point of the CCD is shown in Figure S1. In addition to the spiked target VOCs, numerous background VOCs originating from the virgin PP matrix were detected, resulting in a highly complex chromatographic profile. Therefore, to accurately monitor the 27 target VOCs, their peak areas were extracted using the previously established retention times and characteristic quantifier ions listed in Table 1. The peak areas for each VOC were then normalized to ensure consistency and comparability in subsequent statistical analyses. Specifically, for each VOC, the maximum peak area observed across all experimental conditions was set to 1, and the peak areas obtained under the remaining conditions were expressed as relative values with respect to this maximum. The normalized responses of the target VOCs were then subjected to CCD-based response surface analysis, and individual quadratic models were established for each compound. The optimal extraction conditions for each VOC were subsequently calculated based on the fitted model parameters, and the results are summarized in Table 4. Overall, the CCD-based quadratic models provided satisfactory fitting for most detectable VOCs, as indicated by generally high model R^2^ and adjusted R^2^ values, together with large model parameter F values. Most compounds showed model R^2^ values above 0.90. Meanwhile, lower predicted R^2^ values were observed mainly for several low-vapor-pressure VOCs, suggesting weaker predictive capability and less stable extraction responses for these compounds.

**Table 3:** Coded and Actual Values of Independent Variables for the 1st Rotatable CCD

Factor	Variable	Unit	–α (−1.682)	–1	0	+1	+α (+1.682)
A	SPME temperature	°C	60.00	72.16	90.00	107.84	120.00
B	Incubation time	min	3.00	4.62	7.00	9.38	11.00
C	Extraction time	min	3.00	4.62	7.00	9.38	11.00

**Table 4:** First CCD Optimization Results of 24 Detectable VOCs in the Temperature Range of 60-120 °C

						Significant factors					Optimized condition
No.	Chemical name	model *R* ^2^	Adjust *R* ^2^	Predict *R* ^2^	Parameters F value	1	2	3	SPME Temperature (°C)	Incubation time (min)	Extraction time (min)
1	2,4-dimethyl-1-heptene	0.9923	0.9854	0.9481	143.84	A, C		A^2^	60.5	3.3	3.1
2	1,3-diethylbenzene	0.9966	0.9936	0.9767	328.16	A, C		A^2^	60.3	4.7	3.2
3	*p*-cymene	0.9978	0.9958	0.9942	499.28	A, C		A^2^	61.0	10.6	3.6
4	toluene	0.9923	0.9854	0.9708	143.2	A, B		A^2^	60.1	6.0	3.3
5	*m*-xylene	0.9968	0.994	0.9844	348.4	A, C		A^2^	60.1	7.6	4.9
6	2-ethylhexanal	0.9946	0.9897	0.9639	204.51	A, C		A^2^	60.1	8.4	7.0
7	ethyl acetate	0.9599	0.9283	0.875	26.58	A, B		A^2^	60.6	10.3	10.3
8	1-butanol	0.9846	0.9707	0.9447	70.92	A, B		A^2^	60.2	8.7	4.0
9	ethyl butanoate	0.9879	0.977	0.9158	90.77	A		A^2^	60.0	8.2	3.2
10	1-decanol	0.901	0.812	0.6737	10.12	A		A^2^	78.8	7.6	9.1
11	*n*-pentadecane	0.9768	0.9559	0.8304	46.78	A, C		A^2^	107.2	3.9	7.3
12	BHT	0.9894	0.9799	0.9265	103.76	A, B, C	AC	A^2^	61.3	5.9	10.8
13	tetrahydrolinalool	0.9816	0.965	0.8677	59.27	A			62.2	4.6	9.3
14	ethyl salicylate	0.989	0.9792	0.9394	100.25	A, B, C	AC	A^2^	80.2	5.1	10.7
15	γ-decalactone	0.8903	0.7915	0.3222	9.01	A, C		A^2^	102.7	5.0	10.5
16	5-methylfurfural	0.9854	0.9723	0.9484	75.11	A			60.4	9.1	8.3
17	pentanamide	0.8879	0.7871	0.3869	8.8	A, C		A^2^	95.4	7.0	7.6
18	myristic acid	0.9957	0.9918	0.9755	256.53	A, B, C	AC	A^2^	119.6	9.3	7.0
19	lindane	0.8051	0.6298	0.4324	4.59	B, C			108.9	3.7	8.4
20	ethylene brassylate	0.9691	0.9413	0.786	34.86	A		A^2^	120.0	4.6	6.9
21	BHA	0.8303	0.6776	0.266	5.44	B, C	AB	A^2^	90.3	3.6	10.7
22	ε-caprolactam	0.9243	0.8562	0.5377	13.57	A		A^2^	70.5	10.9	10.5
23	4-(4-hydroxyphenyl)-2-butanone	0.8281	0.6733	–0.2141	5.35	A		A^2^	118.5	5.0	7.0
24	dimethoate	0.8473	0.7099	–0.1568	6.17	A		A^2^	117.6	3.3	10.0
25	High and medium VP VOCs (17)	0.9897	0.9805	0.9636	107.16	A			61.5	8.3	9.9
26	Low VP VOCs (7)	0.9241	0.8558	0.5547	13.53	A, B, C	AB		119.4	6.7	10.4

SPME temperature (A) and its quadratic term (A^2^) were the most frequently significant factors, highlighting the dominant influence of temperature on HS-SPME extraction efficiency. The optimized SPME temperature generally increased as VOC volatility decreased. Extraction time (C) was the next most frequently significant factor, followed by incubation time (B), suggesting that both time-related parameters contributed to VOC extraction but generally showed weaker effects than temperature. Significant interaction terms were observed for several VOCs, particularly AC and AB, indicating that the influence of SPME temperature may interact with the incubation or extraction duration for specific VOCs.

Since recycled plastics often contain complex VOC matrices, analyzing each VOC under its individually optimized conditions may be time-consuming and unnecessary, particularly for readily detectable compounds. In practice, using a limited number of optimized conditions to monitor multiple VOCs is more feasible. Because analyte volatility substantially affected the optimized SPME temperature, which was the most influential factor, the selected VOCs were divided into two categories: (1) high- and medium-vapor-pressure VOCs with relatively low optimized SPME temperatures (Groups 1–6) and (2) low-vapor-pressure VOCs requiring elevated SPME temperatures (Groups 7–9). The normalized responses within each category were summed to generate composite response variables. CCD analysis was then performed using these composite responses, and the optimization results are also presented in Table 4. For high- and medium-vapor-pressure VOCs, the optimized SPME conditions were an SPME temperature of 61.5 °C, incubation time of 8.3 min, and extraction time of 9.9 min. For low-vapor-pressure VOCs, the optimized conditions were an SPME temperature of 119.4 °C, incubation time of 6.7 min, and extraction time of 10.4 min. Therefore, the 24 detectable VOCs can be monitored using two analytical runs under the corresponding optimized conditions.

However, further examination of the optimized parameters showed that the optimal SPME temperature for the high- and medium-vapor-pressure VOC category, as well as for many individual high-vapor-pressure VOCs, was close to the lower boundary of the CCD design. This suggests that the first RSM experiment may not have fully captured the temperature effect for these compounds and that the true optimum may lie below the investigated temperature range. Therefore, a second RSM experiment using a lower SPME temperature range of 30–90 °C was conducted to refine the optimized SPME conditions for this category, as discussed in Section 3.4.

In contrast, the optimal SPME temperature for the low-vapor-pressure VOC category, as well as for many individual low-vapor-pressure VOCs, was close to the upper boundary of the CCD design. In addition, as indicated by Figure 2 and the significant model factors in Table 4, SPME temperature was the dominant factor affecting SPME extraction efficiency for these VOCs. Therefore, additional single-factor experiments focused on SPME temperature were performed to further refine and optimize the extraction conditions for this category, as discussed in Section 3.5.

**Figure 2:**
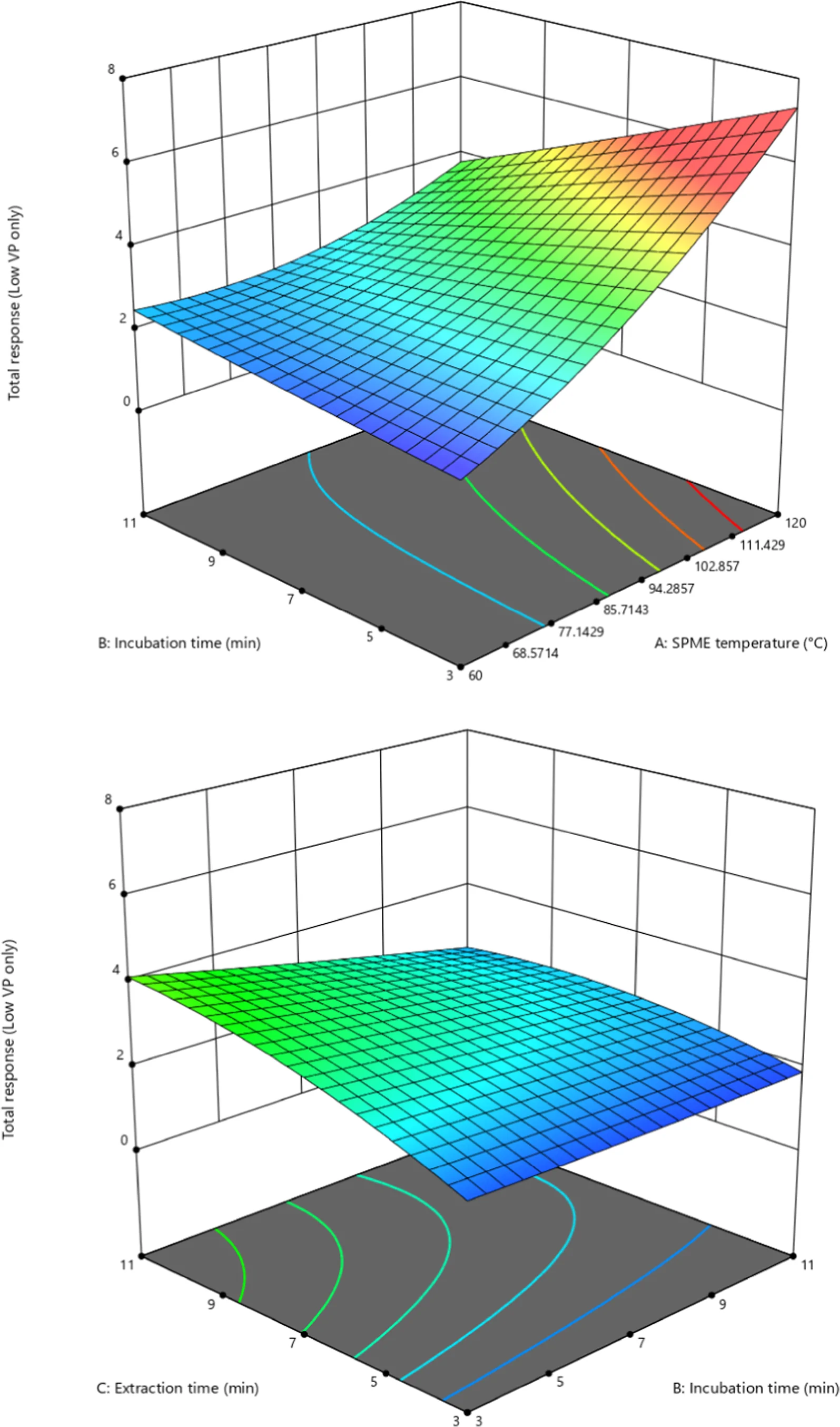
Response surface plots for the low-vapor-pressure VOC category.

### 3.4 Optimization of HS-SPME-GC-MS Conditions for High- and Medium-Vapor-Pressure VOCs

A second set of RSM experiments was conducted using a lower SPME temperature range of 30–90 °C to further optimize the SPME conditions for VOCs in the high- and medium-vapor-pressure category. The CCD design is detailed in Table 5, and the optimization results are summarized in Table 6. The developed quadratic models generally showed good fitting performance, with most compounds exhibiting satisfactory model R^2^, adjusted R^2^, and model parameter F values. Although the predicted R^2^ values varied among compounds, the overall model performance supported their use for optimization within the investigated design space. Compared with the first CCD, the second CCD provided a more appropriate design space for high- and medium-vapor-pressure VOCs by extending the lower temperature boundary. Notably, seven of the nine high-vapor-pressure VOCs showed optimized SPME temperatures below the lower boundary of the first CCD design (60 °C), confirming that the second CCD more effectively captured the temperature-dependent extraction behavior of relatively volatile compounds at lower temperatures.

**Table 5:** Coded and Actual Values of Independent Variables for the 2nd Rotatable CCD

Factor	Variable	Unit	–α (−1.682)	–1	0	+1	+α (+1.682)
A	SPME temperature	°C	30.00	42.16	60.00	77.84	90.00
B	Incubation time	min	3.00	4.62	7.00	9.38	11.00
C	Extraction time	min	3.00	4.62	7.00	9.38	11.00

**Table 6:** CCD Optimized Results of High- and Medium-Vapor-Pressure VOCs From the Temperature Range 30-90 °C

						Significant factors			Optimized condition		
No	Indexes	model *R* ^2^	Adjust *R* ^2^	Predict *R* ^2^	Parameters *F* value	1	2	3	SPME Temperature (°C)	Incubation time (min)	Extraction time (min)
1	2,4-dimethyl-1-heptene	0.914	0.8366	0.364	11.81	A		A^2^	47.9	5.4	9.7
2	1,3-diethylbenzene	0.8879	0.7871	0.1772	8.8	A, C		A^2^	69.6	7.8	8.9
3	*p*-cymene	0.9699	0.9429	0.7988	35.86	A, C	AC	A^2^	63.8	4.0	10.4
4	toluene	0.9482	0.9016	0.6477	20.34	A		A^2^	30.4	7.6	11.0
5	*m*-xylene	0.9387	0.8835	0.6061	17	A, C		A^2^	54.8	3.5	11.0
6	2-ethylhexanal	0.89	0.7909	0.2277	8.99	A		A^2^	57.9	7.0	8.6
7	ethyl acetate	0.9965	0.9934	0.9781	317.41	A, C		A^2^	30.1	10.4	10.6
8	1-butanol	0.9761	0.9547	0.8379	45.47	A		A^2^	33.9	10.4	5.4
9	ethyl butanoate	0.9335	0.8737	0.5438	15.6	A		A^2^	38.3	3.0	11.0
10	1-decanol	0.9762	0.9548	0.8313	45.6	A, C			85.7	7.5	10.7
11	*n*-pentadecane	0.9873	0.9758	0.9053	86.17	A, C	AC	A^2^	88.8	5.1	7.2
12	BHT	0.9896	0.9803	0.9267	106.13	A, C	AB, AC	A^2^	73.8	5.3	10.3
13	tetrahydrolinalool	0.9926	0.9859	0.9479	148.41	A, C	AB, AC	A,^2^ C^2^	73.2	3.6	9.1
14	ethyl salicylate	0.9708	0.9445	0.8049	36.93	A, C		A^2^	90.0	6.0	7.8
15	γ-decalactone	0.9807	0.9634	0.8921	56.55	A, C	AC		82.6	6.0	10.1
16	5-methylfurfural	0.9447	0.8949	0.6405	18.98	A, C		A^2^	89.5	9.3	10.5
17	pentanamide	0.9551	0.9147	0.7108	23.63	A, C		A^2^	84.7	3.4	9.1
18	High and medium VP VOCs (17)	0.9576	0.9194	0.7892	25.08	A, C		A^2^	68.7	5.5	10.4
19	High VP VOCs (9)	0.9538	0.9121	0.7178	22.92	A, C		A^2^	47.9	6.5	9.4
20	Medium VP VOCs (8)	0.9911	0.983	0.9444	123.17	A, C	AC		87.7	10.9	7.7

To establish practical category-level extraction conditions, composite responses were generated for the overall high- and medium-vapor-pressure VOC category, as well as for the high-vapor-pressure and medium-vapor-pressure subcategories. The composite responses were used for regression modeling. The resulting models showed good fitting performance, indicating that the composite-response approach provided reliable optimized conditions for simultaneous VOC detection. For routine broad screening, the overall optimized conditions for the whole category offer a practical compromise that allows multiple VOCs to be monitored in a single analytical run. However, when the analytical objective is focused on a more specific volatility range, the subcategory-specific optimized conditions may be more appropriate, as they better reflect the distinct extraction behaviors of high-vapor-pressure and medium-vapor-pressure VOCs and may improve extraction efficiency for the target group.

Among the three factors investigated, SPME temperature (A) was identified as the most influential parameter, as indicated by its consistent significance across the models. In contrast, incubation time (B) had a limited effect, whereas extraction time (C) showed a moderate influence for selected compounds, sometimes through interaction terms with temperature (AC) and, in one case, through its quadratic term (C^2^). As shown in the response surface plots for the high- and medium-vapor-pressure VOC category (Figure 3), SPME temperature dominated the extraction behavior, while the time-related parameters contributed to fine-tuning extraction efficiency.

**Figure 3:**
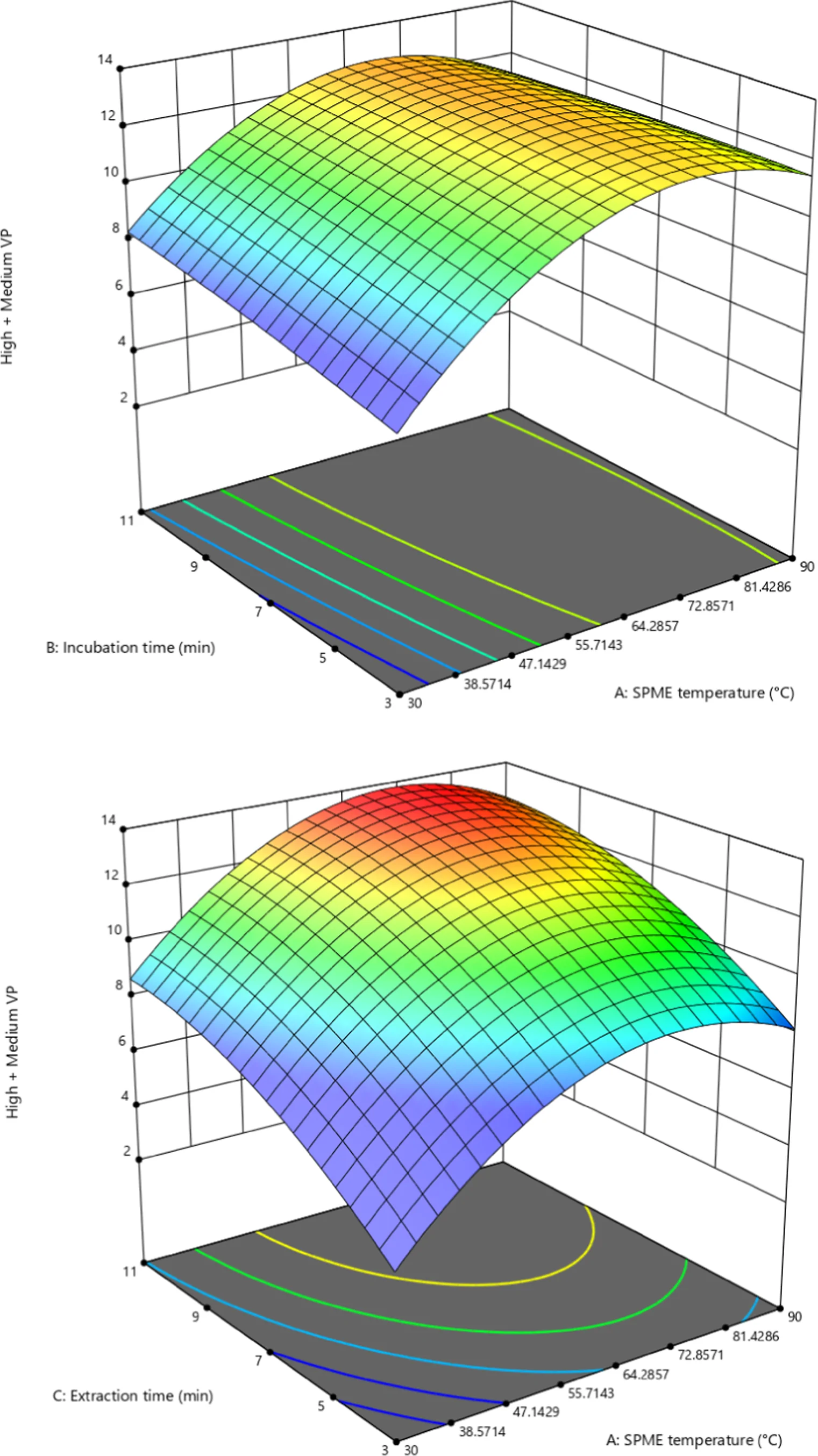
Response surface plots for the high- and medium-vapor-pressure VOC category obtained from the second CCD.

Temperature affects HS-SPME extraction through competing kinetic and thermodynamic effects.^13^ From a kinetic perspective, increasing temperature accelerates mass transfer from the matrix to the headspace and subsequently to the fiber, allowing extraction to proceed more rapidly. Thermodynamically, however, increasing temperature can reduce the analyte-fiber coating affinity constant and, consequently, the fiber-headspace partition coefficient, thereby decreasing analyte loading on the fiber and promoting desorption. Therefore, the optimal SPME temperature reflects a balance between the two effects. In general, the optimal temperature increased as compound volatility decreased. For high-vapor-pressure VOCs, transfer to the headspace is already favorable at relatively low temperatures; so, the thermodynamic reduction in fiber uptake becomes dominant at lower temperatures, resulting in optimal temperatures mainly within the range of 30–60 °C. Medium-vapor-pressure VOCs require greater thermal input to enhance mass transfer from the PP matrix, shifting their optimal temperatures toward approximately 70–90 °C.

Correlation analysis was conducted to examine the relationships between VOC physicochemical properties, including logP and vapor pressure, and the corresponding optimized HS-SPME conditions. Since vapor pressure values spanned several orders of magnitude, direct use of raw vapor pressure could cause the correlation analysis to be disproportionately influenced by highly volatile compounds. In addition, volatilization and headspace partitioning are thermodynamically related to logarithmic pressure terms. Therefore, logarithmically transformed vapor pressure (log VP) was used for the correlation analysis. The resulting scatterplot matrix is shown in Figure 4, and the correlation coefficients with corresponding p-values are summarized in Table 7.

**Figure 4:**
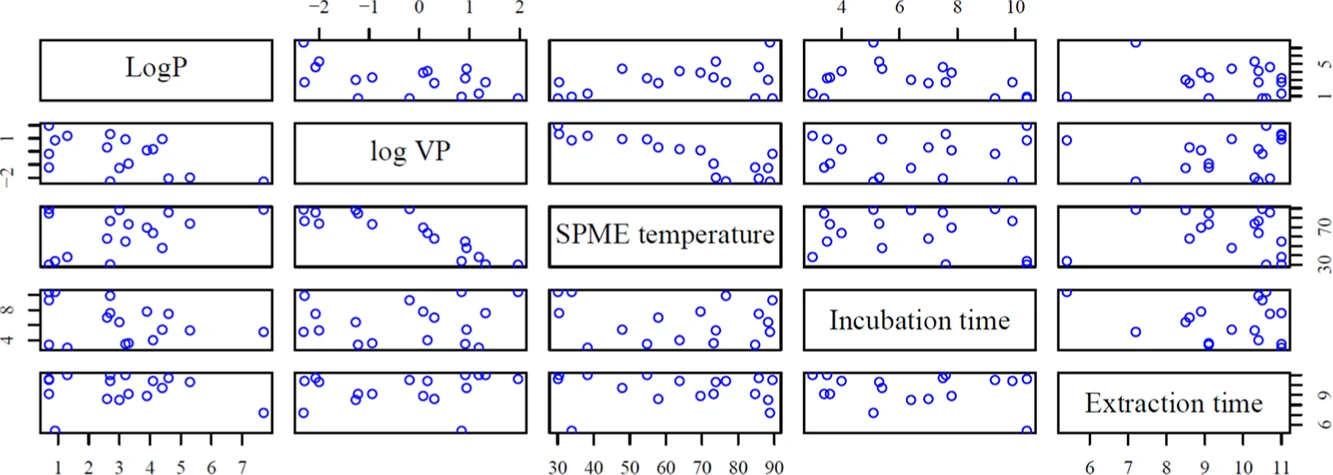
Scatterplot matrix showing relationships between VOC physicochemical properties and optimized HS-SPME conditions.

**Table 7:** Correlation Matrix of VOC Physicochemical Properties and Optimized HS-SPME Conditions

	Log*P*	Log VP^a^	SPME temperature	Incubation time	Extraction time
Log*P*	1				
Log VP	–0.5181 (0.0331)^b^	1			
SPME temperature	0.3758 (0.1371)	–0.8609 (0.0000)	1		
Incubation time	–0.3148 (0.2184)	0.1042 (0.6908)	–0.1720 (0.5092)	1	
Extraction time	–0.09490 (0.7171)	0.1527 (0.5584)	–0.07729 (0.7681)	–0.1869 (0.4727)	1

a Note: Log VP denotes the logarithmic value of VOC vapor pressure.

b Values in parentheses represent *p*-values.

A strong negative correlation was observed between log VP and optimized SPME temperature (r = – 0.8609, *p* < 0.001). Among the correlations between VOC physicochemical properties and optimized HS-SPME conditions, this was the only statistically significant relationship, indicating that VOC volatility was the primary physicochemical factor governing the optimized extraction temperature. Linear regression was further performed between log VP and optimized SPME temperature (Figure 5). The resulting equation was T_SPME_ = −13.197 × (log VP) + 60.42 (R^2^ = 0.7411, n = 17), where T_SPME_ is the optimized SPME temperature in °C and log VP represents the base-10 logarithm of vapor pressure in mmHg. The 95% confidence intervals were −17.49 to −8.90 for the slope and 54.48 to 66.36 for the intercept. Although all 17 observations were included in the regression, the relationship was established using a limited number of compounds. Therefore, the equation can only be used to approximate the SPME temperature of a target VOC based on its vapor pressure. In addition, category-level SPME temperatures can also be estimated by applying the regression equation to the average log VP values of the included VOCs. Using this approach, the estimated SPME temperatures for the high-vapor-pressure subcategory, medium-vapor-pressure subcategory, and combined high-/medium-vapor-pressure category were 49.1, 80.7, and 64.0 °C, respectively. These values were close to the corresponding RSM-optimized temperatures of 47.9, 87.8, and 68.7 °C, supporting the usefulness of log VP as a practical indicator for selecting or approximating suitable SPME temperatures.

**Figure 5:**
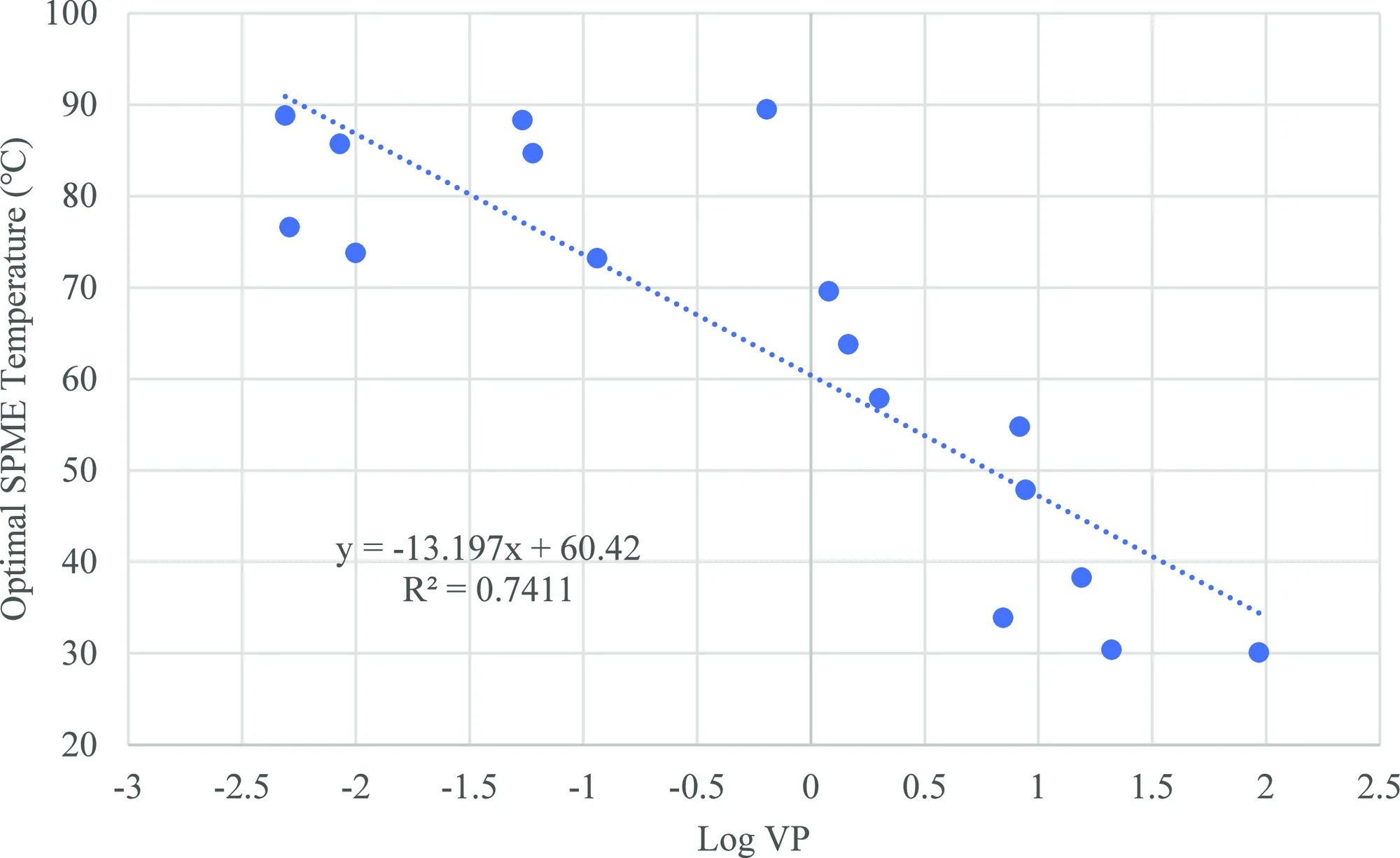
Relationship between optimized SPME temperature and log-transformed vapor pressure for detected high- and medium-vapor-pressure VOCs.

### 3.5 Optimization of the SPME Temperature for Low-Vapor-Pressure VOCs

Because SPME temperature was identified as the dominant factor affecting the extraction of low-vapor-pressure VOCs, a single-factor experiment was performed to further optimize the SPME temperature for this compound category. The SPME temperature was varied from 70 to 150 °C, while the incubation and extraction times were both maintained at 7.0 min. As shown in Figure 6, the responses of most low-vapor-pressure VOCs initially increased with increasing temperature and then decreased at higher temperatures. The increase in response from 70 °C indicates that elevated temperature promoted the release of low-volatility VOCs from the PP matrix into the headspace. Maximum or near-maximum responses were generally observed within the range of 100–120 °C, although the optimal temperature varied among individual compounds. The initial increase in response between 70 °C and approximately 100–120 °C can be attributed to the combined increase in analyte vapor pressure and acceleration of diffusion and mass transfer from the PP matrix into the headspace. For these low-vapor-pressure compounds, the resulting increase in analyte availability initially outweighed the concurrent reduction in fiber uptake. At higher temperatures, however, the decrease in the analyte-fiber coating affinity constant and the consequent reduction in the fiber-headspace partition coefficient, together with enhanced analyte desorption from the fiber, became dominant, causing the response to decline. Notably, 110 °C provided consistently high responses across the low-vapor-pressure VOCs, even when the maximum response of some individual compounds occurred at slightly lower or higher temperatures. Therefore, 110 °C could be selected as a suitable compromise SPME temperature for simultaneous analysis of this compound category. These results further confirm that low-vapor-pressure VOCs generally require higher SPME temperatures than high- and medium-vapor-pressure VOCs to achieve efficient HS-SPME extraction. Although no obvious deterioration in fiber performance was observed during the limited number of experiments conducted in this study, repeated use of the DVB/C-WR/PDMS fiber at 110 °C may accelerate coating aging and increase background signals over time. Therefore, fiber performance and blank responses should be monitored periodically when the low-vapor-pressure method is used routinely, and the fiber should be replaced if evidence of reduced extraction efficiency or increased background is observed.

**Figure 6:**
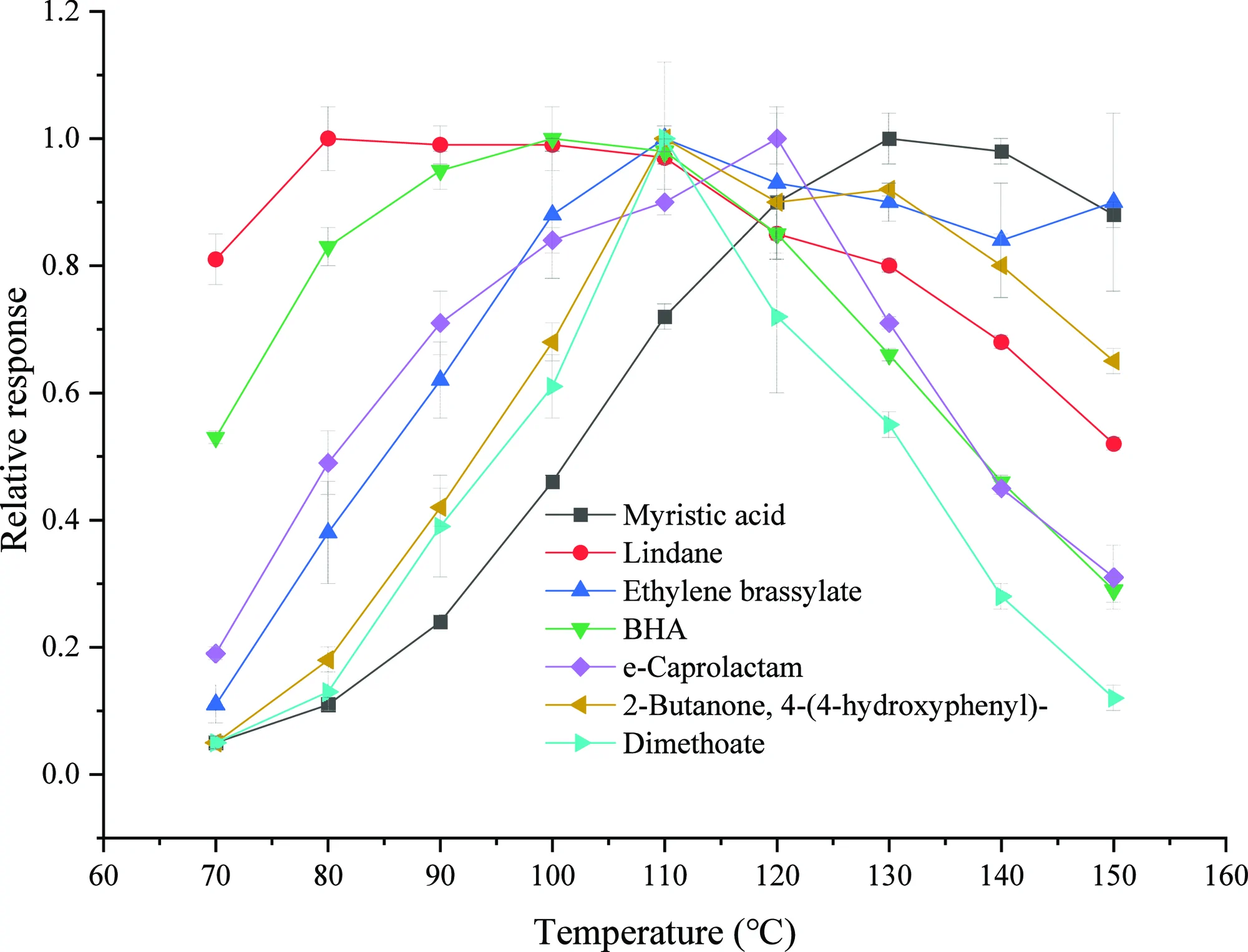
Effect of SPME temperature on the extraction efficiency of low-vapor-pressure VOCs.

### 3.6 Applicability of the HS-SPME-GC-MS Method to Industrial Recycled PP

Four industrial recycled PP samples, including mixed-color and natural-color materials collected before and after pelletization, were analyzed under the two optimized HS-SPME conditions established for the high-/medium-vapor-pressure and low-vapor-pressure VOC categories (Figure 7). A representative TIC obtained under the optimized conditions for the high- and medium-vapor-pressure VOC category is shown in Figure S2. Similar to what was observed for the PP model samples, the recycled PP sample exhibited a highly complex VOC profile. Therefore, the characteristic quantifier and qualifier ions of each target VOC were examined within the corresponding previously established retention-time window to ensure accurate detection. Several VOCs, including 2,4-dimethyl-1-heptene, *p*-cymene, toluene, m-xylene, 1-decanol, n-pentadecane, tetrahydrolinalool, ethyl salicylate, and lindane, were detected in one or more recycled PP samples (Table 8). These results demonstrate the applicability of the developed HS-SPME-GC-MS method for VOC screening in real recycled plastic matrices. Since this study was designed as a qualitative screening method and no calibration was performed, the peak areas reported in Table 8 represent instrument responses only and should not be used for quantitative or direct comparisons among different VOCs or samples. Since no analyte-specific calibration was conducted, limits of detection (LODs) and limits of quantification (LOQs) were not determined in this work. Accordingly, the sensitivity of the method at concentrations relevant to health-based or regulatory thresholds remains to be established and represents a limitation of the present study.

**Figure 7:**
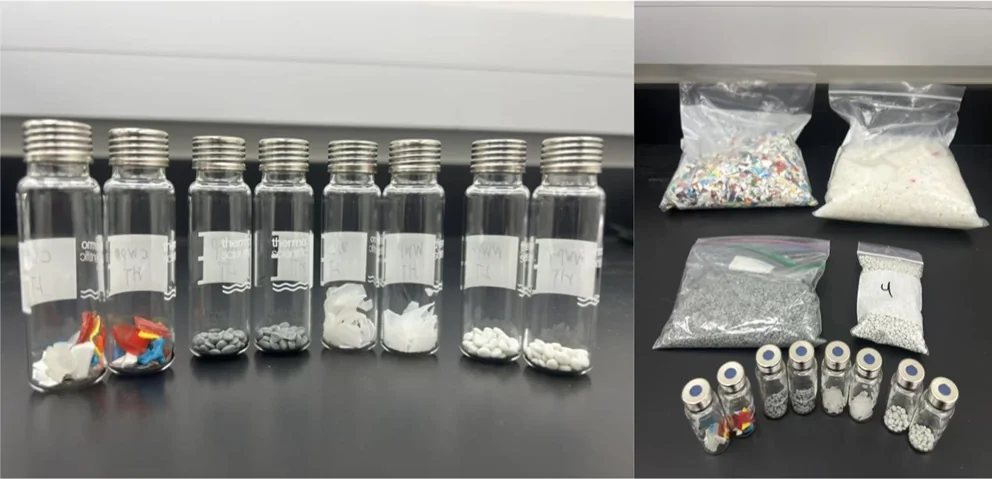
Industrial recycled PP samples analyzed in this study.

**Table 8:** Chromatographic Peak Areas of VOCs Identified in Recycled PP Samples^a^

VOC	MB	MA	NB	NA
2,4-dimethyl-1-heptene	1693872	1403019	1512071	28528093
*p*-cymene	330586	1859814	152300	24964512
toluene	127284	120479	88134	374392
*m*-xylene	234335	171571	120154	1618579
1-decanol	217575	199379	272067	269781
*n*-pentadecane	40246556	29299455	2118690	33843839
tetrahydrolinalool	179869	2248510	4075862	3502813
ethyl salicylate	289684	225967	ND	341359
lindane	ND	ND	29579	ND

a Note: MB: mixed-color rPP before pelletization; MA: mixed-color rPP after pelletization; NB: natural-color rPP before pelletization; NA: natural-color rPP after pelletization; ND, not detected.

The detected VOCs likely originated from multiple sources associated with the life cycle and reprocessing of plastics. Hydrocarbons such as 2,4-dimethyl-1-heptene and n-pentadecane may be related to thermo-oxidative or mechanical degradation of PP, in which polymer chain scission can generate low-molecular-weight branched alkenes and alkanes. Aromatic compounds, including toluene, m-xylene, and *p*-cymene, may be associated with external contamination sources such as printing inks, solvents, adhesives, or absorbed residues from previous product use. Oxygenated compounds, including 1-decanol, tetrahydrolinalool, and ethyl salicylate, are more plausibly associated with consumer-product residues, such as fragrances, flavoring agents, surfactants, detergents, or personal-care products. The occurrence of these compounds suggests that part of the recycled PP stream may have originated from packaging or consumer products previously exposed to scented materials, cleaning agents, or personal-care formulations. Lindane was the only low-vapor-pressure VOC detected in the recycled PP samples and was observed only in the natural-color sample before pelletization. Its presence may indicate residual contamination from pesticide-related containers. Since only a single aliquot of each recycled PP sample was analyzed under each optimized condition, replicate variability and the potential effects of sample heterogeneity were not evaluated. Therefore, the recycled-sample results should be interpreted as a qualitative demonstration of method applicability.

## 4 Conclusions

This study established a property-guided HS-SPME-GC-MS workflow for the qualitative screening of potentially hazardous VOCs in recycled PP. From 622 candidate compounds, 367 substances were classified as potentially hazardous based on NFPA health hazard ratings, and 274 compounds with sufficient volatility were retained as relevant VOC candidates. Twenty-seven representative target compounds were then selected across vapor-pressure and polarity domains, enabling method development to reflect the broad physicochemical diversity of the VOCs expected in real recycled plastic materials. Among the five SPME fiber coatings evaluated, DVB/C-WR/PDMS provided the broadest overall coverage, detecting 24 of the 27 representative VOCs, and was therefore selected for method optimization.

Response surface methodology was used to optimize key HS-SPME parameters. SPME temperature was identified as the dominant factor controlling extraction efficiency, and VOC vapor pressure was the primary physicochemical parameter associated with optimal SPME temperature. High- and medium-vapor-pressure VOCs generally exhibited reduced responses at elevated temperatures, whereas low-vapor-pressure VOCs required higher temperatures to enhance transfer from the PP matrix to the headspace. These contrasting behaviors indicate that two separate extraction conditions are necessary for broad VOC screening. The optimized condition for high- and medium-vapor-pressure VOCs was an SPME temperature of 68.7 °C, incubation time of 5.5 min, and extraction time of 10.4 min. For low-vapor-pressure VOCs, 110 °C was identified as a suitable compromise SPME temperature when the incubation and extraction times were fixed at 7.0 min.

Application of the optimized method to industrial recycled PP samples demonstrated its applicability, with hydrocarbons, aromatic compounds, oxygenated compounds, and lindane detected in real recycled PP. Overall, the developed method provides a practical qualitative screening approach for characterization of potentially hazardous VOCs in recycled PP and can support quality control, emission assessment, and process optimization in plastic recycling systems.

## Supplementary Material











## Notes

Representative TICs obtained from a PP model sample spiked with the 27 representative VOCs and from an industrial recycled PP sample (Figures S1 and S2) (PDF)
potential contaminants in recycled plastics (XLSX)
potentially hazardous VOCs in recycled plastics (XLSX)
detailed information on the representative VOCs (XLSX)
evaluation results for the representative VOCs using different SPME fibers (XLSX)
