# Supplementary material for: Development of a Property-Guided HS-SPME-GC-MS Method for Broad-Range Qualitative Screening of Potentially Hazardous VOCs in Recycled Polypropylene
Source: ACS Polym Au. 2026 Aug 24;6(5):1422–34. doi: 10.1021/acspolymersau.6c00124 (PMC13673883; doi:10.1021/acspolymersau.6c00124)
Supplement: Supplementary file 1 [file lg6c00124_si_001.pdf]

Supporting Information

Development of a Property-Guided HS-SPME-GC-MS Method for Broad-Range Qualitative Screening of Potentially Hazardous VOCs in Recycled Polypropylene

Wei Jiang[1], Huijun Yi[2], Caleb Chatfield[1], Chloe Higdon[1], Nathan Mancos[1], Shaoyang Liu[1,*]

[1] Center for Materials and Manufacturing Sciences, Departments of Chemistry and Physics, Troy University, Troy, AL, 36082 United States

[2] Department of Mathematics and Statistics, Troy University, AL 36082, USA

* Corresponding Author: lius@troy.edu

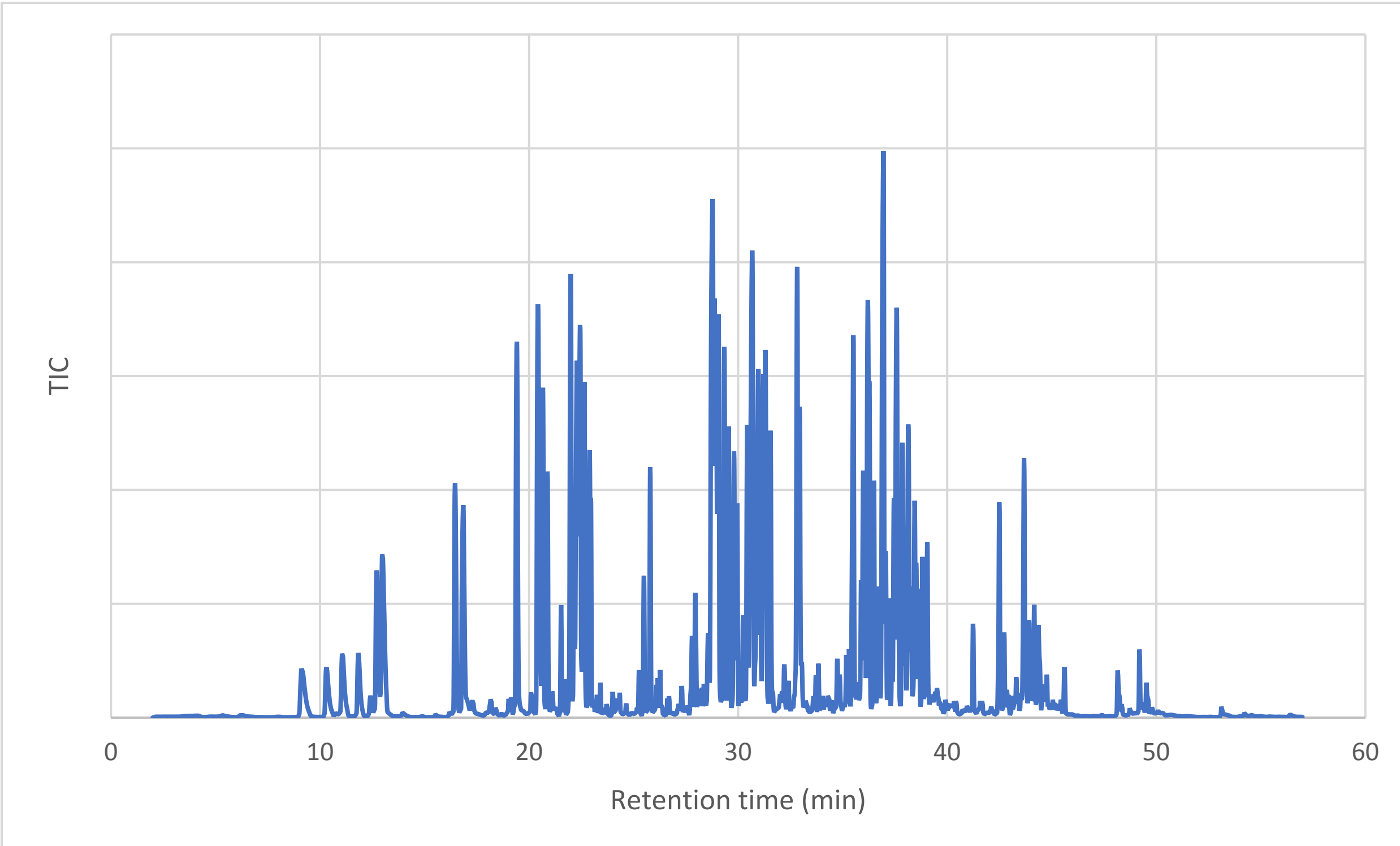

Figure S1. Representative total ion chromatogram (TIC) obtained from a PP model sample spiked with the 27 representative VOCs (SPME fiber: DVB/C-WR/PDMS; SPME temperature: 90 °C; incubation time: 7 min; extraction time: 7 min).

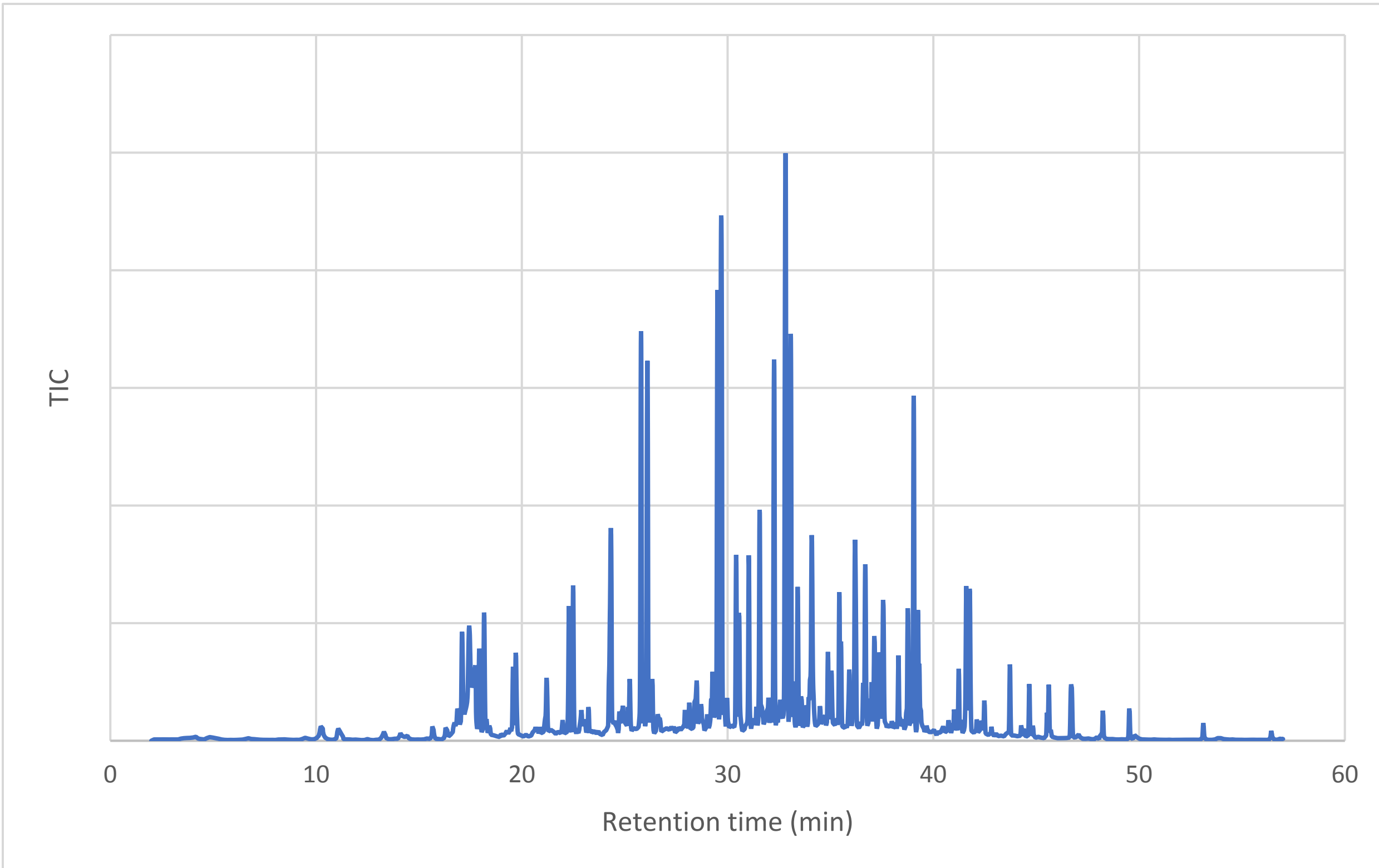

Figure S2. Representative total ion chromatogram (TIC) obtained from an industrial recycled PP sample (SPME fiber: DVB/C-WR/PDMS; SPME temperature: 68.7 °C; incubation time: 5.5 min; extraction time: 10.4 min).
